# Bullying and Cyberbullying Are Associated with Low Levels of Motivational Beliefs Toward Learning in Youth

**DOI:** 10.3390/ejihpe15060093

**Published:** 2025-05-23

**Authors:** Jose Luis Solas-Martínez, Rubén Roldán-Roldán, María de las Nieves Moyano-Muñoz, Emilio J. Martínez-López

**Affiliations:** 1 Department of Didactics of Musical, Plastic and Corporal Expression, University of Jaen, 23071 Jaén, Spain; jsolas@ujaen.es (J.L.S.-M.); emilioml@ujaen.es (E.J.M.-L.); 2 Department of Psychology, University of Jaen, 23071 Jaén, Spain; mnmoyano@ujaen.es (M.d.l.N.M.-M.)

**Keywords:** aggressors, gender differences, motivational beliefs, test anxiety, victims

## Abstract

This study explored the association between bullying and cyberbullying, both in victims and bullies, and motivational beliefs toward learning in students aged 10 to 16. A cross-sectional study was conducted with 1690 Spanish students, assessing motivational beliefs through the Motivated Strategies for Learning Questionnaire (MSLQ) and involvement in bullying using the European Bullying Intervention Project Questionnaire (EBIP-Q) and the European Cyberbullying Intervention Project Questionnaire (ECIP-Q). The results showed that both victims and bullies had lower task value, self-efficacy, and control beliefs, along with higher test anxiety, with cyberbullying having a stronger impact. Victims of cyberbullying exhibited significantly lower task value (up to 9.2% in girls and 5.6% in boys) and had a 4.5- and 2.2-times higher risk of scoring low in this dimension. Among bullies, only girls involved in traditional bullying showed motivational deficits, whereas both male and female cyberbullies had task value scores up to 9.5% lower and were 1.5 to 1.6 times more likely to experience test anxiety. These findings emphasize the need for targeted interventions to reinforce motivational beliefs in victims and bullies, recommending collaborative programs between students, teachers, and families to enhance task value, control beliefs, and self-efficacy while addressing test anxiety.

## 1. Introduction

### 1.1. Motivational Beliefs and Their Role in Academic Engagement

Motivational beliefs are psychological constructs that reflect how students interpret, value, and engage with educational tasks and goals, as well as their expectations regarding their own ability to achieve academic success (Burns et al., 2018; Van Vu et al., 2024). Rather than being a set of static attributes, these beliefs emerge from a dynamic process shaped by personal, contextual, and social factors (Steinmayr et al., 2019). Key components of motivational beliefs include intrinsic and extrinsic goal orientation, perceived task value, sense of control over the learning process, self-efficacy, and test anxiety (Pintrich et al., 1991). These elements are closely interconnected, influencing the quality of student engagement, persistence in the face of challenges, and the effectiveness of problem-solving strategies (Van Vu et al., 2024). As such, the strength of these beliefs is crucial for academic performance and psychological well-being, as it determines the adoption of adaptive or maladaptive attitudes toward learning (Burns et al., 2018).

Motivational beliefs are also a multidimensional construct composed of several variables, among which six key components have been identified as crucial for learning (Pintrich et al., 1991). First, intrinsic goal orientation reflects students’ internal interest and enjoyment in engaging with challenging tasks, fostering deeper comprehension and more self-directed study habits (Liu et al., 2020). In contrast, extrinsic goal orientation is driven by external incentives, such as grades or rewards, which effectively promote short-term achievement (Liu et al., 2020). Another essential component is task value, which refers to students’ perceptions of the importance, usefulness, and interest of a given activity, thereby encouraging more meaningful engagement (Song & Chung, 2020). Additionally, control beliefs pertain to students’ perceived ability to influence academic outcomes through personal effort, fostering a sense of autonomy and guiding the selection of effective learning strategies (Worick et al., 2023). Self-efficacy, defined as confidence in one’s own abilities to successfully complete academic tasks, is closely linked to persistence and resilience (Beatson et al., 2020). Lastly, test anxiety is associated with the emotional distress triggered by evaluation scenarios, which often impairs cognitive functioning and performance (Roos et al., 2023). The absence or weakening of these interconnected motivational components undermines cognitive engagement, restricts adaptive academic behaviors, hinders the application of advanced strategies, and ultimately negatively affects performance and long-term educational trajectories (Kryshko et al., 2020).

However, motivational beliefs can be significantly impacted by adverse social dynamics that are highly prevalent in school environments, such as bullying and cyberbullying (Cañas et al., 2020). The presence of these behaviors exerts a detrimental effect on motivation, well-being, and self-perception, hindering the development of positive motivational beliefs and, consequently, academic success (Cañas et al., 2020; Delgado et al., 2019). Focusing on motivational beliefs is particularly relevant because they represent a proximal determinant of students’ academic behavior, directly influencing engagement, persistence, and performance. In the context of bullying and cyberbullying, these beliefs are often undermined before academic achievement is affected, making them a sensitive indicator of psychological and educational impact (Aparisi et al., 2021). Moreover, motivational beliefs are modifiable through intervention, which makes them an actionable target for prevention and support programs (Samara et al., 2021).

From a theoretical perspective, motivation research offers important insights into how experiences such as bullying and cyberbullying might affect students’ academic functioning. According to the Self-Determination Theory, students’ learning motivation is fundamentally driven by the satisfaction of three basic psychological needs: autonomy (the need to feel control over one’s actions), competence (the need to feel effective and capable), and relatedness (the need to feel connected to others) (Urhahne & Wijnia, 2023). When these needs are thwarted by adverse social experiences, such as bullying and cyberbullying, students may experience diminished intrinsic motivation, reduced academic engagement, and increased emotional distress (Cañas et al., 2020). These processes are directly linked to motivational components such as self-efficacy (reflecting perceived competence), control beliefs (related to autonomy), and test anxiety (as an emotional response to perceived failure or lack of control) (Liu et al., 2023; Pintrich et al., 1991). Similarly, Expectancy–Value Theory proposes that motivation is determined by two key cognitive factors: the expectation of success in a given task and the subjective value placed on that task (Koenka, 2020). In this study, these constructs are operationalized through the variables, task value and self-efficacy, both of which are susceptible to decline in the presence of social stressors such as victimization or aggression (Delgado et al., 2019; Liu et al., 2023). Negative experiences in the school environment, particularly victimization or involvement in aggressive behavior, can reduce both the perceived likelihood of success and the importance attributed to academic activities, thereby impairing motivational beliefs and engagement (Delgado et al., 2019). These theoretical frameworks thus provide the foundation for the present study’s hypotheses, which anticipate that involvement in bullying or cyberbullying will be negatively associated with these motivational beliefs, with variations based on sex and type of involvement.

### 1.2. Bullying, Cyberbullying, and Their Impact on Academic Motivation

Bullying is defined as repeated, intentional aggression directed by one or more students toward a peer perceived as vulnerable, manifesting in physical, verbal, or relational forms (Galán-Arroyo et al., 2024). According to Hosozawa et al. (2021), the global prevalence of bullying among adolescents is approximately 30.4%, with the most common forms being verbal (21.4%), relational (20.9%), and physical (15.2%) aggression. Cyberbullying, on the other hand, involves the use of digital platforms to harass, humiliate, or threaten individuals and is more prevalent in specific psychological environments, disproportionately affecting girls (Vismara et al., 2022). Unlike traditional bullying, cyberbullying can infiltrate all aspects of a victim’s private life and provide bullies with a sense of anonymity and impunity (Bork-Hüffer et al., 2020). Furthermore, in certain contexts, global prevalence rates of cyberbullying have been reported as high as 46.3% among bullies and 57.5% among victims (Zhu et al., 2021). Recent studies emphasize that the dynamics of cyberbullying have continued to evolve rapidly with the widespread use of new digital platforms and social media applications among adolescents, intensifying their impact on academic and psychological outcomes (Deol & Lashai, 2022; Rejeb et al., 2024). These negative dynamics significantly impact both academic and psychological functioning, disrupting critical cognitive and emotional processes essential for effective learning (Peled, 2019).

Recent studies have shown that victims tend to exhibit lower levels of engagement, poorer academic performance, and reduced motivation, while their confidence in their own abilities is significantly weakened (L. Li et al., 2020). This loss of self-assurance leads to a diminished valuation of tasks, the adoption of maladaptive beliefs, and a decline in persistence and willingness to face challenges (Obregón-Cuesta et al., 2022; Samara et al., 2021). Over time, these dynamics create a vicious cycle, as low academic performance reinforces negative self-perceptions and limits educational aspirations (Samara et al., 2021). On the other hand, the role of a bully is often associated with distorted perceptions of the importance of effort and cooperative work, fostering a less positive view of the school environment (Aparisi et al., 2021; Montero-Carretero et al., 2020). Although this perspective may not immediately translate into poor academic performance, it gradually undermines motivation, diminishing resilience in the face of difficulties and reducing the willingness to engage in more challenging tasks (Yang & Wang, 2022). In the long term, these distortions hinder the adoption of effective learning strategies and the development of consistent study habits, ultimately leading to poor academic performance (Thorsen et al., 2021).

In addition to the dynamics stemming from bullying, the literature has identified various biological and social factors that significantly influence students’ academic performance and psychological well-being (Poh et al., 2019). In this regard, age affects students’ ability to navigate complex learning and social situations (El Zaatari & Maalouf, 2022; Urruticoechea et al., 2021). Similarly, higher levels of maternal education enhance access to cultural resources, parental support for academic activities, and parental expectations, ultimately leading to better academic outcomes (El Zaatari & Maalouf, 2022; Fateel et al., 2021; Tantoh, 2023). Body mass index (BMI) has also been linked to nutritional and physical imbalances that impair cognitive functioning, concentration, and the energy required for academic tasks (Wassenaar et al., 2021). Likewise, physical activity enhances psychological well-being and improves stress management, positively impacting motivation and engagement in learning (Raine et al., 2020). These factors could mediate or moderate the relationship between bullying experiences and motivational beliefs, influencing the connection between victimization, motivation, and academic performance (Samara et al., 2021).

### 1.3. Gender Differences and Research Gaps

Additionally, gender differences play a crucial role in these dynamics. While boys are more likely to be both victims and bullies, often resorting to threats and physical violence, girls tend to be more vulnerable to psychological bullying (Cosma et al., 2022). In the academic domain, girls exhibit greater self-regulation, employ more effective motivational and self-assessment strategies, cooperate more frequently, and tend to evaluate their performance more positively, fostering the development of stronger motivational beliefs. Conversely, boys, despite demonstrating greater skills in concentration and information processing, show lower self-regulation and are more inclined toward specific performance-oriented goals (Wirthwein et al., 2020). Finally, boys tend to respond more effectively to high-pressure situations, whereas girls excel in maintaining a methodical and consistent approach (Montolio & Taberner, 2021).

The impact of bullying and cyberbullying in educational settings has been extensively examined from the perspective of victims (Aparisi et al., 2021; Obregón-Cuesta et al., 2022). However, the influence of these dynamics on bullies has received significantly less attention (Montero-Carretero et al., 2020). This gap in the literature limits a comprehensive understanding of the effects of school bullying and its interaction with key variables such as motivational beliefs toward learning. Moreover, while it is well established that victimization affects academic performance and motivation (Samara et al., 2021), there are still gaps in precisely quantifying its impact and in developing predictive risk models. The lack of specific tools to measure the degree of victim exposure and its differential influence by gender on academic performance hinders the design of evidence-based preventive strategies. In this context, the present study offers an innovative approach by simultaneously examining the impact of bullying and cyberbullying on both victims and bullies, providing a detailed assessment of the academic risk associated with these experiences. The integration of gender-based differential analyses and the quantification of their effects on motivational beliefs toward learning represent a significant contribution to the field of education. Additionally, given the rapid evolution of digital platforms and the intensifying psychological and academic consequences associated with cyberbullying, addressing these dynamics within a contemporary adolescent population is particularly timely and necessary. This study expands the available knowledge and lays the groundwork for the development of data-driven preventive interventions.

### 1.4. Objectives and Hypotheses of This Study

Based on the above, this study aims to (1) analyze the association between victimization and aggression in bullying and cyberbullying and motivational beliefs toward learning in boys and girls; (2) quantify differences in motivational beliefs between involved and non-involved adolescents; and (3) estimate the risk associated with experiences of bullying and cyberbullying. Accordingly, the main research question guiding this study is how are traditional bullying and cyberbullying experiences associated with adolescents’ academic motivational beliefs, and how do these associations and risks differ by gender? This study contributes to the existing literature by jointly analyzing the effects of both bullying and cyberbullying on motivational beliefs, considering not only the role of victims but also that of aggressors, and providing a detailed examination of gender-based differences. In addition to describing these associations, this study quantifies the motivational differences between those involved in these experiences and their non-involved peers and identifies the relative risk of low academic motivation associated with being a victim or an aggressor. This comprehensive approach allows for a more nuanced understanding of how different forms of school violence impact academic motivation.

## 2. Methods

### 2.1. Participants

A total of 1690 Spanish children and adolescents (829 boys, 49.05%) from seven educational institutions participated in this cross-sectional quantitative study. Participants were students aged 10 to 16 years (M = 13.05, SD = 1.79). Schools were selected based on convenience sampling, with four urban schools (>10,000 inhabitants) and three rural schools (<10,000 inhabitants). Within each school, students were selected using a randomized full-group sampling method, ensuring balanced participation across all institutions. Anthropometric and sociodemographic characteristics are presented in Table 1.

Boys reported higher levels of weekly physical activity and greater involvement in aggressive behaviors compared to girls (*p* < 0.001 and *p* = 0.001, respectively). However, girls experienced higher levels of victimization compared to boys (*p* = 0.014). Additionally, girls scored significantly higher in maternal educational level (*p* < 0.001), academic performance (*p* = 0.001), and four motivational beliefs toward learning: extrinsic goal orientation, task value, self-efficacy, and test anxiety (all *p* < 0.037).

### 2.2. Measures

#### 2.2.1. Dependent Variables: Motivational Beliefs

Learning strategies were assessed using the “Motivated Strategies for Learning Questionnaire” (MSLQ, Pintrich et al., 1991). This self-report instrument consists of 81 items grouped into 15 subscales, designed to evaluate both motivational beliefs related to course content and the use of various learning strategies. For the present study, only the motivational belief section of the questionnaire was used, which includes 31 items. These items form a total of six subscales: (1) intrinsic goal orientation, (2) extrinsic goal orientation, (3) task value, (4) control of learning beliefs, (5) self-efficacy for learning and performance, and (6) test anxiety. This section assesses students’ goals and value beliefs regarding a course, their beliefs about their ability to succeed, and their level of test anxiety in an academic setting. Responses were recorded using a 7-point Likert scale (1 = Totally false for me; 7 = Totally true for me). The reliability of all subscales used in this study was acceptable, with Cronbach’s α ranging from 0.77 to 0.89.

In intrinsic goal orientation, higher scores reflect task engagement driven by challenge, curiosity, and a desire to master content, whereas in extrinsic goal orientation, high scores indicate a focus on obtaining grades, rewards, or external recognition. The task value subscale assesses interest in, importance of, and perceived usefulness of the course, where high scores suggest a positive valuation of academic content. The control of learning belief subscale measures the attribution of success to personal effort, with high scores indicating a greater sense of control over academic outcomes. The self-efficacy for learning and performance subscale evaluates students’ confidence in their ability to understand and complete tasks successfully, where high scores are associated with greater academic self-assurance. Finally, the test anxiety subscale examines cognitive worry and emotional arousal levels, where higher scores indicate interferences that negatively affect academic performance. Overall, high scores on the assessed subscales are beneficial for learning, except in the case of test anxiety, where elevated values represent a detrimental factor (Pintrich et al., 1991).

#### 2.2.2. Predictor/Independent Variables: Bullying and Cyberbullying

The level of bullying was assessed using the “European Bullying Intervention Project Questionnaire”, Spanish version (Ortega-Ruiz et al., 2016), which consists of 14 items. The reliability results were acceptable (Cronbach’s α for victimization = 0.83; Cronbach’s α for aggression = 0.79). To assess cyberbullying, the Spanish version of the “European Cyberbullying Intervention Project Questionnaire” (ECIPQ; Del Rey et al., 2015) was used, comprising 22 items. Reliability results for this instrument were also acceptable (α for cyber-victimization = 0.87; α for cyber-aggression = 0.82). Both questionnaires distinguish two dimensions (victimization and aggression) and employ a Likert-type scale ranging from 1 = never to 5 = more than once a week. Low scores indicate minimal experiences or involvement in bullying or cyberbullying, while high scores reflect frequent experiences of victimization or aggression. Both questionnaires were administered individually, requiring approximately 15 min to complete. The items explore the frequency of these behaviours over the past two months.

#### 2.2.3. Confounding Variables: Age, Maternal Education Level, Body Mass Index, and Weekly Physical Activity Level

Each participant’s age and maternal education level were recorded using a sociodemographic questionnaire. Age was considered a confounding variable due to its relevance in previous studies, which have demonstrated that cognitive and emotional maturity significantly influence how individuals learn and interact with their environment (El Zaatari & Maalouf, 2022; Urruticoechea et al., 2021). Similarly, academic performance, mental health, and intelligence quotient have been found to be significantly associated with maternal education level (Baharvand et al., 2021).

Physical activity level was included as a covariate, as recent research highlights its influence on cognitive development and academic performance in students (D. Li et al., 2023; Petrigna et al., 2022). Additionally, both BMI and physical activity are associated with physical and mental well-being, learning, and self-esteem, and may therefore mediate the effectiveness of learning strategies (Bacon & Lord, 2021; Seum et al., 2022). BMI was calculated using Quetelet’s formula: weight (kg)/height^2^ (m). Weight and height measurements were obtained using a digital scale (ASIMED^®^ Type B, Class III) and a portable stadiometer (SECA^®^ 214, SECA Ltd., Hamburg, Germany). Measurements were taken with light clothing and without shoes.

Weekly physical activity level was assessed using the “PACE+ Adolescent Physical Activity Measure” (Prochaska et al., 2001). This instrument consists of two items in which participants report the number of days they engaged in at least 60 minutes of moderate-to-vigorous physical activity during the past seven days and during a typical week. The final score was calculated as the average of both responses: (R1 + R2)/2. The reliability index for this instrument was α = 0.79.

### 2.3. Procedure

Before data collection, parents, teachers, and school administrators were informed about the purpose of this study, and informed consent was obtained from parents or legal guardians. Each participant’s name was coded to ensure anonymity and confidentiality. Measurements were conducted during school hours, as facilitated by the participating institutions, and the questionnaires were completed in the students’ usual classroom environment under the supervision of researchers and classroom tutors. This study was approved by the Bioethics Committee of the University of Jaén (Spain), reference NOV.22/2.PRY, and its design complies with Spanish regulations on clinical research involving human participants (Law 14/2007, of July 3, on Biomedical Research), data protection laws concerning personal information (Organic Law 15/1999), and the principles of the Declaration of Helsinki (2013, Brazil).

### 2.4. Statistical Analysis

The comparison of continuous and categorical variables between boys and girls was performed using Student’s *t*-test and χ^2^ tests, respectively. The normality and homoscedasticity of the data were verified using the Kolmogorov–Smirnov and Levene tests, respectively. Prior to the analyses, the dataset was screened for missing values. The proportion of missing data was very low (less than 1%), and its randomness was statistically confirmed using Little’s MCAR test (*p* > 0.05). Therefore, missing data were handled using listwise deletion to maintain internal consistency in the inferential analyses. To examine whether adolescents who had never experienced victimization or aggression on bullying or cyberbullying had better motivational beliefs toward learning than those who had been victims or bullies, an analysis of covariance (ANCOVA) was conducted. Each motivational belief (intrinsic goal orientation, extrinsic goal orientation, task value, control of learning beliefs, self-efficacy, and test anxiety) was used as a dependent variable, while bullying victimization, cyberbullying victimization, bullying aggression, and cyberbullying aggression were included as fixed factors. Bullying and cyberbullying values were dichotomized such that participants who reported never being victims or bullies of bullying and/or cyberbullying (questionnaire score = 1) were labeled “Never”, while those who had experienced victimization or aggression at least once (questionnaire score > 1) were labeled “Sometimes”. Since many comparison groups had unequal sample sizes, effect size was calculated using Hedges’ g, with 0.2 = small effect, 0.5 = medium effect, and 0.8 = large effect (Martínez-López et al., 2018). The percentage of difference between groups was calculated as follows: [(Large-measurement − Small-measurement)/Small-measurement] × 100. To determine the risk level associated with bullying and cyberbullying victimization/aggression in developing lower motivational beliefs toward learning, a binary logistic regression analysis was performed. For this purpose, dependent variables were dichotomized using the median as a reference (Kwon et al., 2020; Lepinet et al., 2023). For each motivational belief, participants were classified as high (≥ median; reference group) vs. low (< median; risk group). In all analyses, age, BMI, maternal education level, and weekly physical activity were included as covariates. Analyses were conducted separately for boys and girls, with a 95% confidence level (*p* < 0.05) applied to all statistical tests. All computations were performed using SPSS v. 25.0 for Windows (SPSS Inc., Chicago, IL, USA).

## 3. Results

### 3.1. ANCOVA Analysis of Bullying and Cyberbullying Victimization in Relation to Motivational Beliefs Toward Learning

**Figure 1:**
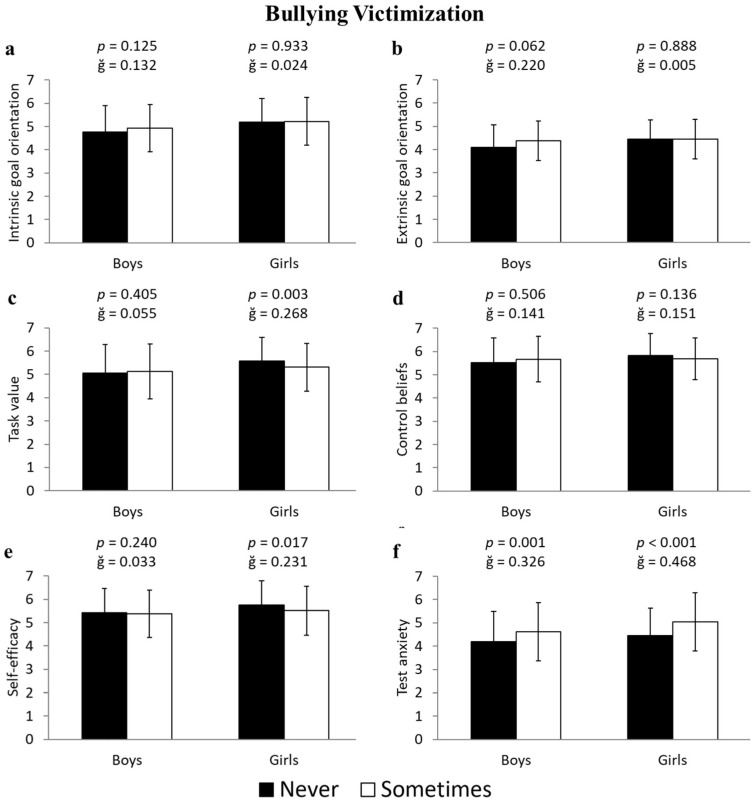
Differences in the subscales of motivational beliefs toward learning—(**a**) intrinsic goal orientation, (**b**) extrinsic goal orientation, (**c**) task value, (**d**) control beliefs, (**e**) self-efficacy, and (**f**) test anxiety—between students who have never been victims and those who have experienced bullying victimization, differentiated by boys and girls.

Girls who were victims of bullying showed statistically lower scores compared to non-victimized girls in task value (−5.2%; 5.31 ± 1.02 vs. 5.58 ± 1.02 u.a.; F(1,643) = 9.600, *p* = 0.003, ğ = 0.268, 1-β = 0.834) and self-efficacy (−4.4%; 5.51 ± 1.05 vs. 5.75 ± 1.05 u.a.; F(1,643) = 5.760, *p* = 0.017, ğ = 0.231, 1-β = 0.669), as well as higher scores in test anxiety (11.5%; 5.04 ± 1.25 vs. 4.46 ± 1.17 u.a.; F(1,643) = 17.621, *p* < 0.001, ğ = 0.468, 1-β = 0.987) (Figure 1c, Figure 1e, and Figure 1f, respectively). Similarly, boys who were victims of bullying had higher scores in test anxiety (8.9%; 4.61 ± 1.25 vs. 4.2 ± 1.28 u.a.; F(1,596) = 11.643, *p* = 0.001, ğ = 0.236, 1-β = 0.926) compared to their non-victimized peers (Figure 1f).

**Figure 2:**
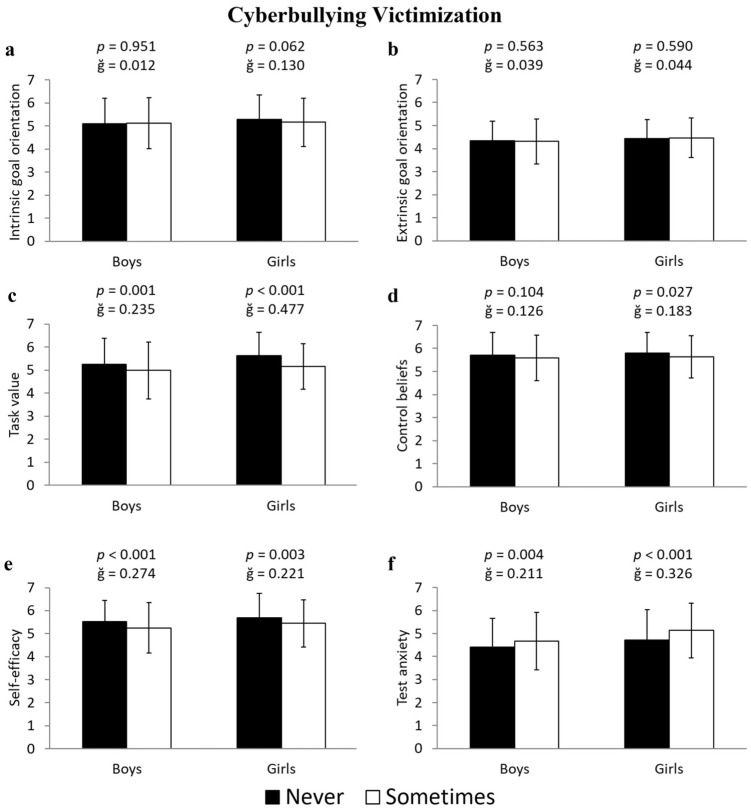
Differences in the subscales of motivational beliefs toward learning—(**a**) intrinsic goal orientation, (**b**) extrinsic goal orientation, (**c**) task value, (**d**) control beliefs, (**e**) self-efficacy, and (**f**) test anxiety—between students who have never been victims and those who have experienced cyberbullying victimization, differentiated by boys and girls.

Girls who were victims of cyberbullying had lower scores than non-victimized girls in task value (−9.2%, 5.63 ± 1.00 vs. 5.15 ± 0.99 u.a.; F(1,643) = 38.453, *p* < 0.001, ğ = 0.477, 1-β < 0.999), control beliefs (−2.9%, 5.80 ± 0.89 vs. 5.63 ± 0.91 u.a.; F(1,643) = 4.885, *p* = 0.027, ğ = 0.183, 1-β = 0.598), and self-efficacy (−4.3%, 5.68 ± 1.07 vs. 5.45 ± 1.03 u.a.; F(1,643) = 9.048, *p* = 0.003, ğ = 0.221, 1-β = 0.852), as well as higher scores in test anxiety (7.9%, 4.73 ± 1.31 vs. 5.13 ± 1.20 u.a.; F(1,643) = 15.420, *p* < 0.001, ğ = 0.326, 1-β = 0.975) (Figure 2c, Figure 2d, Figure 2e, and Figure 2f, respectively). For boys who were victims of cyberbullying, the data revealed lower scores in task value (−5.6%, 5.26 ± 1.13 vs. 4.98 ± 1.24 u.a.; F(1,596) = 11.427, *p* = 0.001, ğ = 0.235, 1-β = 0.921) and self-efficacy (−5.3%, 5.53 ± 0.92 vs. 5.25 ± 1.10 u.a.; F(1,596) = 12.813, *p* < 0.001, ğ = 0.274, 1-β = 0.947), along with higher scores in test anxiety (5.7%, 4.40 ± 1.26 vs. 4.67 ± 1.26 u.a.; F(1,596) = 8.570, *p* = 0.004, ğ = 0.211, 1-β = 0.832) (Figure 2c, Figure 2e, and Figure 2f, respectively).

### 3.2. ANCOVA Analysis of Bullying and Cyberbullying Aggression in Relation to Motivational Beliefs Toward Learning

**Figure 3:**
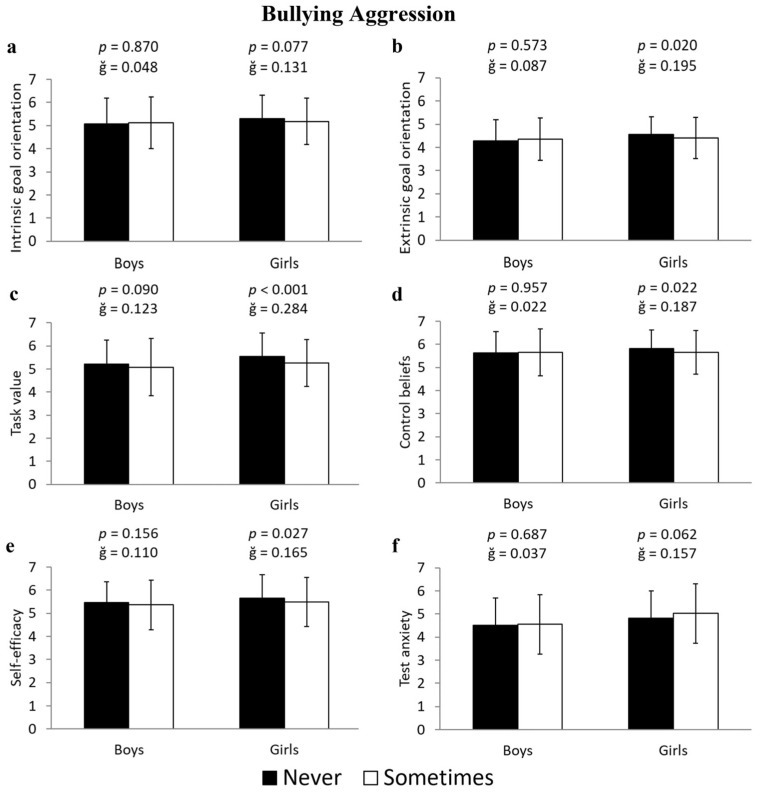
Differences in the subscales of motivational beliefs toward learning—(**a**) intrinsic goal orientation, (**b**) extrinsic goal orientation, (**c**) task value, (**d**) control beliefs, (**e**) self-efficacy, and (**f**) test anxiety—between students who have never engaged in bullying aggression and those who have, differentiated by boys and girls.

Girls who were bullies had significantly lower scores in four of the motivational belief factors: extrinsic goal orientation (−3.7%; 4.57 ± 0.74 vs. 4.40 ± 0.88 u.a.; F(1,643) = 5.479, *p* = 0.020, ğ = 0.195, 1-β = 0.647), task value (−5.5%; 5.55 ± 1.01 vs. 5.26 ± 1.02 u.a.; F(1,643) = 15.150, *p* < 0.001, ğ = 0.284, 1-β = 0.973), control beliefs (−3.0%; 5.82 ± 0.79 vs. 5.65 ± 0.94 u.a.; F(1,643) = 5.306, *p* = 0.022, ğ = 0.187, 1-β = 0.633), and self-efficacy (−3.2%; 5.66 ± 1.00 vs. 5.49 ± 1.07 u.a.; F(1,643) = 4.886, *p* = 0.027, ğ = 0.165, 1-β = 0.598) (Figure 3b, Figure 3c, Figure 3d, and Figure 3e, respectively). However, boys who were bullies showed no significant differences in motivational beliefs toward learning compared to non-bullies (all *p* > 0.05, Figure 3).

**Figure 4:**
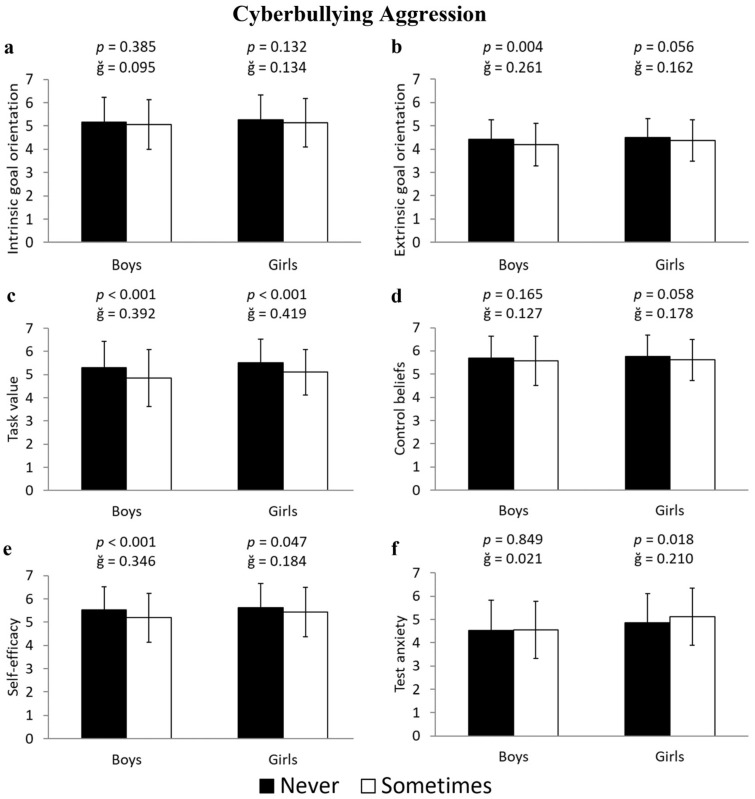
Differences in the subscales of motivational beliefs toward learning—(**a**) intrinsic goal orientation, (**b**) extrinsic goal orientation, (**c**) task value, (**d**) control beliefs, (**e**) self-efficacy, and (**f**) test anxiety—between students who have never engaged in cyberbullying aggression and those who have, differentiated by boys and girls.

The data also showed that girls who were cyberbullying bullies had lower scores in task value (−8.3%; 5.52 ± 1.02 vs. 5.10 ± 0.99 u.a.; F(1,643) = 23.102, *p* < 0.001, ğ = 0.419, 1-β = 0.998) and self-efficacy (−3.6%; 5.62 ± 1.04 vs. 5.43 ± 1.06 u.a.; F(1,643) = 3.961, *p* = 0.047, ğ = 0.243, 1-β = 0.511), as well as higher scores in test anxiety (5.2%; 4.86 ± 1.26 vs. 5.12 ± 1.24 u.a.; F(1,643) = 5.600, *p* = 0.018, ğ = 0.210, 1-β = 0.657) (Figure 4c, Figure 4e, and Figure 4f, respectively). Similarly, boys who were cyberbullying bullies had lower scores in extrinsic goal orientation (−5.7%, 4.4 ± 0.82 vs. 4.20 ± 1.00 u.a.; F(1,596) = 8.165, *p* = 0.004, ğ = 0.261, 1-β = 0.814), task value (−9.5%, 5.31 ± 1.13 vs. 4.85 ± 1.23 u.a.; F(1,596) = 18.915, *p* < 0.001, ğ = 0.392, 1-β = 0.991), and self-efficacy (−6.7%, 5.54 ± 0.97 vs. 5.18 ± 1.06 u.a.; F(1,596) = 15.657, *p* < 0.001, ğ = 0.346, 1-β = 0.977) (Figure 4b, Figure 4c, and Figure 4e, respectively).

### 3.3. Binary Logistic Regression Analysis of Bullying and Cyberbullying Victimization in Relation to Motivational Beliefs Toward Learning

The results indicating the risk of exposure to bullying and cyberbullying victimization concerning motivational beliefs toward learning are presented in Table 2. Girls who were victims of bullying were 1.75 times more likely than non-victimized girls to have low task value (Odds Ratio [OR] = 1.750, *p* < 0.001) and 1.7 times more likely to obtain high scores in test anxiety (OR = 1.749, *p* < 0.001). Similarly, boys who were victims of bullying had a 1.54 times higher risk of low task value (OR = 1.542, *p* < 0.001) and a 1.29 times higher risk of low self-efficacy (OR = 1.293, *p* = 0.024) compared to non-victimized boys. Additionally, girls who were victims of cyberbullying were significantly more likely than non-victimized girls to have lower scores in task value (OR = 4.499, *p* < 0.001), control beliefs (OR = 1.650, *p* = 0.021), and self-efficacy (OR = 1.697, *p* = 0.016). For boys, the analysis revealed a significantly increased risk of task value (OR = 2.244, *p* < 0.001) and self-efficacy (OR = 1.954, *p* = 0.002).

### 3.4. Binary Logistic Regression Analysis of Bullying and Cyberbullying Aggression in Relation to Motivational Beliefs Toward Learning

The results indicating the risk of exposure to bullying and cyberbullying aggression concerning motivational beliefs toward learning are presented in Table 3. Girls who were bullies were 1.42, 2.08, and 1.38 times more likely than non-bullies to have low scores in extrinsic goal orientation (OR = 1.419, *p* = 0.017), task value (OR = 2.075, *p* < 0.001), and control beliefs (OR = 1.383, *p* = 0.029), respectively. For boys who were bullies, significant risks were found for low task value (OR = 1.813, *p* < 0.001), control beliefs (OR = 1.464, *p* = 0.011), and self-efficacy (OR = 1.574, *p* = 0.003). Additionally, girls who were cyberbullies had a higher probability of low task value (OR = 1.734, *p* = 0.001) and a greater risk of high test anxiety (OR = 1.628, *p* = 0.003). The analysis for boys who were cyberbullies revealed a significant risk for low task value (OR = 1.466, *p* = 0.031). 

## 4. Discussion

### 4.1. Main Findings

The primary objective of this study was to analyze the association between bullying and cyberbullying victimization/aggression and motivational beliefs toward learning in children and adolescents aged 10 to 16 years. The results revealed that, regardless of whether students were victims or bullies of bullying and/or cyberbullying, all forms of harassment were negatively associated with motivational beliefs toward learning, particularly among girls. Table 4 presents a summary of the data, differentiating the motivational beliefs of victims and bullies of bullying and cyberbullying, along with the percentage decreases/increases and the corresponding risk increments.

### 4.2. Interpretation of Results and Comparison with Previous Literature

Our results indicate that bullying and cyberbullying are negatively associated with motivational beliefs toward learning in both victims and bullies, particularly by reducing task value and self-efficacy, as well as significantly increasing test anxiety. This effect is especially pronounced in victims of traditional bullying, where test anxiety is 11.5% higher compared to non-victimized peers. These findings align with previous studies demonstrating that both victims and bullies of bullying and cyberbullying exhibit higher levels of anxiety and depression, which negatively affect their engagement in learning and perceived academic competence (Eyuboglu et al., 2021). The decline in task value and self-efficacy is associated with lower confidence in academic abilities, leading to reduced motivation and poorer academic performance (Liu et al., 2023). Notably, our data suggest that the negative association of cyberbullying is broader and more severe than that of traditional bullying. Recent studies have highlighted those cyberbullying victims experience greater disruptions in academic self-concept and achievement goals (Delgado et al., 2019). Furthermore, persistent and heightened social anxiety in these students significantly compromises their ability to manage academic stress, implement effective learning strategies, and adapt to academic demands (Aparisi et al., 2021; Martínez-Monteagudo et al., 2020).

The present study also found that girls who were victims exhibited greater motivational deficits than girls who were bullies, particularly in cyberbullying, with notably lower scores in task value and self-efficacy, as well as higher levels of test anxiety. These findings are consistent with previous studies indicating that girls tend to experience greater academic anxiety and a lower perception of competence compared to boys (Rusillo-Magdaleno et al., 2024). Additionally, a lack of clarity in self-concept and a greater tendency toward fatalism among female cyberbullying victims contribute to a more negative perception of their academic abilities and reduced motivation for learning (Geng et al., 2022). On the other hand, only boys who were victims exhibited negative associations similar to those observed in boys who were bullies, in both bullying and cyberbullying. A review of the literature indicates that both victims and bullies of bullying and cyberbullying may struggle with emotional self-regulation and stress management (Geng et al., 2022). Additionally, research has shown that both victims and bullies in bullying situations tend to exhibit high levels of ruminative anger and diminished impulse control (Zsila et al., 2019). These factors may contribute to the persistence of aggressive behaviors, negatively affecting motivation and academic performance. 

Regarding gender differences, our results indicate that girls exhibit greater vulnerability in the association between bullying and cyberbullying and their motivational beliefs, showing more pronounced differences compared to their non-victimized peers than those observed in boys. This aligns with previous reviews indicating that adolescent girls who are victims of bullying and cyberbullying experience a more significant decline in academic self-concept and achievement goals compared to boys (Delgado et al., 2019). Additionally, research has found that in girls, the relationship between victimization and low academic performance is mediated by higher levels of depressive symptoms, whereas in boys, this relationship is less pronounced (Okumu et al., 2020). These differences may be attributed to sociocultural factors that shape how girls respond to bullying. It appears that adolescent girls tend to experience greater social pressure to maintain positive interpersonal relationships, thereby increasing their perceived emotional harm from bullying and cyberbullying (Okumu et al., 2020). Furthermore, girls are more vulnerable to developing anxiety and depression, as they are more likely to cope with bullying through internalization strategies, which can significantly compromise their motivation and academic self-efficacy (Potard et al., 2022). These findings reinforce the need for gender-sensitive intervention programs, as detailed in the recommendations section.

The findings of this study reveal that cyberbullying victims, both girls and boys, face the greatest risk of low motivational beliefs. Among girls, this risk is 4.5 times higher for task value and 1.7 times higher for self-efficacy and control beliefs, while among boys, the risk reaches 2.2 times for task value and 2 times for self-efficacy. Although traditional bullying victims also face risks, they are comparatively lower, reinforcing the idea that constant exposure to cyberbullying exacerbates the negative effects on motivation (Przybylski & Bowes, 2017). These findings support the notation that cyberbullying is more negatively associated with academic motivation than traditional bullying, aligning with previous studies that highlight the potential of digital harassment to cause prolonged psychological harm and negatively affect perceived academic competence (Aparisi et al., 2021; Delgado et al., 2019). Additionally, both bullying and cyberbullying bullies face an increased risk of scoring low in task value, along with a 1.4 and 1.7 times higher risk of scoring low in control beliefs and self-efficacy. Also, cyberbullying girls show a greater risk of high test anxiety. These results suggest that the role of a bully also carries negative consequences for academic motivation, possibly due to a lower valuation of learning and weaker academic commitment (Rusillo-Magdaleno et al., 2024). The findings presented in this study not only reinforce previous evidence but also contribute novel insights by quantifying the magnitude of the issue, illustrating how these students may be influenced by impulsive behavioral patterns and the normalization of aggression in their social environment, ultimately reducing their motivation for learning and their willingness to develop effective strategies (Cañas et al., 2020; Valera-Pozo et al., 2021). Taken together, these findings provide strong evidence that cyberbullying represents a greater threat to academic motivation than traditional bullying, particularly due to its broader and more severe associations with reduced task value, control beliefs, and self-efficacy, as well as heightened test anxiety. Importantly, girls appear more vulnerable to these negative academic outcomes, especially in the role of victim, whereas boys show more consistent motivational deficits when involved as aggressors. These patterns suggest that interventions must be differentiated not only by type of involvement (victim vs. aggressor) but also by gender and context (cyber vs. traditional settings). Addressing these nuanced risk profiles is critical to mitigating the motivational disengagement that often precedes academic underperformance and dropout in adolescents. 

### 4.3. Recommendations to Strengthen Motivational Beliefs Toward Learning in Bullying and Cyberbullying Contexts

Table 5 presents a series of recommendations aimed at enhancing motivational beliefs toward learning in victims and bullies of bullying and cyberbullying. These recommendations are differentiated based on their application to students, teachers, and families, considering gender differences and the type of aggression. The guidelines presented here are based on both the findings of this study and international reference research (Compton et al., 2014; Hinduja & Patchin, 2017; O’Higgins Norman, 2020). Given the negative association between bullying and academic motivation, it is essential to implement preventive strategies that foster a positive perception of learning and reduce the risk of low self-efficacy, low task value, and test anxiety. To achieve this, interventions should prioritize strengthening confidence and a sense of control over learning in victims, while also developing awareness programs for bullies to promote a safer and more equitable school environment.

### 4.4. Limitations and Strengths

This study presents several methodological limitations that should be acknowledged. First, its cross-sectional design prevents the establishment of causal relationships, as it does not allow for conclusions regarding the direction of the observed effects. Second, the use of self-report measures may introduce biases such as social desirability or inaccurate recall, potentially affecting the accuracy of the reported experiences with bullying and cyberbullying. Third, convenience sampling limits the generalizability of the results, as the findings may not be representative of students from other regions, different socioeconomic backgrounds, or educational systems. Moreover, psychosocial factors such as family support and peer relationship quality were not controlled for in the analyses, which may influence both the experience of bullying and academic motivation. Despite these limitations, this study has several strengths: the use of coding techniques to ensure participant anonymity and confidentiality; the use of highly reliable measures with demonstrated internal validity; and the inclusion of a wide range of relevant covariates, including age, body mass index, maternal education level, and weekly physical activity. These elements enabled the identification of specific risk levels and outcomes that may contribute to meaningful advances in educational research. Finally, although the present study identifies statistical associations between bullying or cyberbullying and motivational beliefs, it does not examine potential mediating psychological mechanisms, such as emotional regulation, self-concept, or social cognition, that may explain how these experiences influence academic motivation. Future research should explore these pathways to provide a deeper understanding of the underlying processes. 

## 5. Conclusions

This study concludes that both victimization and aggression in bullying and cyberbullying are negatively associated with motivational beliefs toward learning in youth aged 10 to 16 years. In traditional bullying, victims exhibit lower scores in task value and self-efficacy, with differences of up to 5.5% in girls and 5.2% in boys, as well as higher test anxiety (11.5% in girls and 8.9% in boys). In the case of cyberbullying, the results are even more concerning than those observed in traditional bullying. Victims display lower task value scores, with differences of 9.2% in girls and 5.6% in boys, and face a 4.5- and 2.2-times greater risk, respectively, of scoring low in this dimension. Lower scores in self-efficacy, control beliefs, and test anxiety were also observed compared to non-victims, with differences ranging from 2.9% to 7.9%. Regarding bullies, significant differences compared to non-bullies were found only in girls, who showed lower scores in extrinsic goal orientation, task value, control beliefs, and self-efficacy. However, both boys and girls exhibited a 1.4- to 2.1-times greater risk of scoring low in task value and control beliefs. In cyberbullying, bullies had lower scores than non-bullies in extrinsic goal orientation, task value, and self-efficacy, with differences ranging from 5.7% to 9.5% in boys and 3.6% to 8.3% in girls. Additionally, both genders showed a 1.5- to 1.6-times higher risk of elevated test anxiety.

These findings highlight the urgent need to implement educational strategies that strengthen motivational beliefs in both victims and bullies. It is recommended to develop programs that promote task value, control beliefs, and self-efficacy, alongside strategies to manage test anxiety. Achieving this requires collaborative efforts among students, teachers, and families, ensuring a school environment that fosters confidence in academic abilities, reduces the emotional impact of bullying, and encourages a positive attitude toward learning. 

## Figures and Tables

**Table 1:** Biometric characteristics and sociodemographic data of participants segmented by gender.

	All (n = 1690)		Boys (n = 829)		Girls (n = 861)		
Variables	Mean	SD/%	Mean	SD/%	Mean	SD/%	*p*
Age (years)	13.05	1.79	13.03	1.81	13.06	1.77	0.759
Weight (kg)	51.81	13.52	53.70	14.95	49.99	11.69	<0.001
Height (m)	1.59	0.11	1.60	0.13	1.57	0.08	<0.001
BMI (kg/m^2^)	20.41	4.00	20.55	3.93	20.26	4.06	0.126
Maternal education level (%)							<0.001
No education		4.5%		4.4%		4.6%	
Primary (EGB)		9.6%		10.4%		8.8%	
Secondary (BUP)		13.0%		11.0%		14.9%	
Professional training		13.5%		13.6%		13.6%	
University		38.2%		34.8%		41.3%	
Do not know		21.2%		25.9%		16.8%	
Weekly physical activity (average)	4.01	1.76	4.30	1.81	3.73	1.67	<0.001
Academic performance	6.92	1.55	6.79	1.53	7.06	1.56	0.001
Bullying Victimization (%)							0.014
Never		14.1%		16.6%		11.6%	
Occasionally		52.0%		48.7%		55.2%	
Once or twice/month		24.4%		24.2%		24.5%	
Once/week		7.0%		7.5%		6.6%	
More than once/week		2.5%		3.0%		2.1%	
Bullying Aggression (%)							0.001
Never		25.9%		24.1%		27.5%	
Occasionally		58.9%		56.8%		59.8%	
Once or twice/month		11.5%		13.5%		9.6%	
Once/week		3.6%		5.1%		2.2%	
More than once/week		0.7%		0.5%		0.9%	
Cyberbullying Victimization (%)							0.091
Never		43.2%		45.8%		40.7%	
Occasionally		50.1%		47.0%		53.1%	
Once or twice/month		5.0%		5.3%		4.7%	
Once/week		1.6%		1.7%		1.5%	
More than once/week		0.1%		0.2%		0.0%	
Cyberbullying Aggression (%)							0.550
Never		57.3%		56.5%		58.2%	
Occasionally		38.0%		38.5%		37.5%	
Once or twice/month		3.1%		3.6%		2.6%	
Once/week		1.6%		1.4%		1.7%	
More than once/week		0.0%		0.0%		0.0%	
Motivational beliefs toward learning							
Intrinsic goal orientation	5.17	1.04	5.13	1.08	5.21	1.01	0.162
Extrinsic goal orientation	4.41	0.87	4.35	0.90	4.46	0.84	0.008
Task value	5.22	1.12	5.16	1.19	5.28	1.05	0.037
Control beliefs	5.68	0.95	5.66	0.99	5.69	0.91	0.516
Self-efficacy	5.45	1.05	5.38	1.03	5.51	1.07	0.013
Test anxiety	4.78	1.29	4.62	1.28	4.94	1.27	<0.001

Note. Data are presented as means for continuous variables and frequency (%) for categorical variables. BMI = Body Mass Index; SD = Standard Deviation.

**Table 2:** Binary logistic regression of bullying and cyberbullying victimization (1 = never to 5 = more than once/week) according to categorised indicators (high vs. low) of motivational orientation for learning in adolescent boys and girls. OR: odds ratio, CI: confidence interval. OR was adjusted for age, BMI (body mass index), maternal education, and weekly physical activity.

		Boys (602)				Girls (649)			
Bullying Victimization		N	*p*	OR	95% CI	N	*p*	OR	95%CI
Intrinsic goal orientation	High	254		1	Referent	309		1	Referent
	Low	348	0.941	0.992	0.798–1.232	340	0.068	0.812	0.650–1.015
Extrinsic goal orientation	High	284		1	Referent	344		1	Referent
	Low	318	0.627	1.055	0.85–1.309	305	0.422	0.914	0.733–1.139
Task value	High	286		1	Referent	342		1	Referent
	Low	316	<0.001	1.542	1.225–1.940	307	<0.001	1.750	1.384–2.213
Control beliefs	High	272		1	Referent	315		1	Referent
	Low	330	0.900	0.986	0.795–1.224	334	0.521	0.931	0.749–1.158
Self-efficacy	High	267		1	Referent	351		1	Referent
	Low	335	0.024	1.293	1.034–1.617	298	0.324	1.120	0.894–1.403
Test anxiety	Low	340		1	Referent	314		1	Referent
	High	262	0.325	1.114	0.899–1.380	335	<0.001	1.749	1.382–2.213
**Cyberbullying victimization**									
Intrinsic goal orientation	High	254		1	Referent	309		1	Referent
	Low	348	0.410	1.177	0.799–1.734	340	0.165	1.322	0.891–1.963
Extrinsic goal orientation	High	284		1	Referent	344		1	Referent
	Low	318	0.151	1.326	0.902–1.950	305	0.146	1.335	0.904–1.971
Task value	High	286		1	Referent	342		1	Referent
	Low	316	<0.001	2.244	1.454–3.462	307	<0.001	4.499	2.593–7.809
Control beliefs	High	272		1	Referent	315		1	Referent
	Low	330	0.859	1.035	0.710–1.508	334	0.021	1.65	1.08–2.523
Self-efficacy	High	267		1	Referent	351		1	Referent
	Low	335	0.002	1.954	1.284–2.974	298	0.016	1.697	1.103–2.610
Test anxiety	Low	340		1	Referent	314		1	Referent
	High	262	0.738	1.066	0.734–1.549	335	0.515	1.133	0.778–1.649

**Table 3:** Binary logistic regression of bullying and cyberbullying aggression (1 = never to 5 = more than once/week) according to categorised indicators (high vs. low) of motivational orientation for learning in adolescent boys and girls. OR: odds ratio, CI: confidence interval. OR was adjusted for age, BMI (body mass index), maternal education, and weekly physical activity.

		Boys (602)				Girls (649)			
Bullying Aggression		N	*p*	OR	95% CI	N	*p*	OR	95% CI
Intrinsic goal orientation	High	254		1	Referent	309		1	Referent
	Low	348	0.064	1.317	0.984–1.762	340	0.206	1.2	0.904–1.593
Extrinsic goal orientation	High	284		1	Referent	344		1	Referent
	Low	318	0.059	1.316	0.990–1.750	305	0.017	1.419	1.065–1.890
Task value	High	286		1	Referent	342		1	Referent
	Low	316	<0.001	1.813	1.330–2.472	307	<0.001	2.075	1.506–2.860
Control beliefs	High	272		1	Referent	315		1	Referent
	Low	330	0.011	1.464	1.092–1.963	334	0.029	1.383	1.033–1.851
Self-efficacy	High	267		1	Referent	351		1	Referent
	Low	335	0.003	1.574	1.162–2.133	298	0.130	1.252	0.936–1.675
Test anxiety	Low	340		1	Referent	314		1	Referent
	High	262	0.597	0.928	0.705–1.222	335	0.498	1.100	0.834–1.451
**Cyberbullying aggression**									
Intrinsic goal orientation	High	254		1	Referent	309		1	Referent
	Low	348	0.388	1.164	0.824–1.645	340	0.279	1.195	0.866–1.648
Extrinsic goal orientation	High	284		1	Referent	344		1	Referent
	Low	318	0.479	1.132	0.803–1.594	305	0.398	1.148	0.834–1.581
Task value	High	286		1	Referent	342		1	Referent
	Low	316	0.031	1.466	1.035–2.077	307	0.001	1.734	1.243–2.418
Control beliefs	High	272		1	Referent	315		1	Referent
	Low	330	0.366	1.171	0.831–1.651	334	0.194	1.236	0.898–1.703
Self-efficacy	High	267		1	Referent	351		1	Referent
	Low	335	0.072	1.376	0.972–1.948	298	0.485	1.125	0.808–1.567
Test anxiety	Low	340		1	Referent	314		1	Referent
	High	262	0.813	1.042	0.738–1.472	335	0.003	1.628	1.183–2.241

**Table 4:** Comparison of mean differences and risk ratios in motivational beliefs related to bullying and cyberbullying among victims and bullies, differentiated by gender.

		Boys		Girls	
		Mean Difference	Risk	Mean Difference	Risk
Victims	Bullying	8.9 Test anxiety	x1.5 < Task value x1.3 < Self-efficacy	−5.2% Task value −4.4% Self-efficacy 11.5% Test anxiety	x1.8 < Task value x1.7 < Test anxiety
	Cyberbullying	−5.6% Task value −5.3% Self-efficacy 5.7% Test anxiety	x2.2 < Task value x2.0 < Self-efficacy	−9.2% Task value −2.9% Control beliefs −4.3% Self-efficacy 7.9 Test anxiety	x4.5 < Task value x1.7 < Control beliefs x1.7 < Self-efficacy
Bullies	Bullying	N/A	x1.8 < Task value x1.5 < Control beliefs x1.6 < Self-efficacy	−3.7% Extrinsic goal orientation −5.5% Task value −3.0% Control beliefs −3.2% Self-efficacy	x1.4 < Extrinsic goal orientation x2.1 < Task value x1.4 < Control beliefs
	Cyberbullying	−5.7% Extrinsic goal orientation −9.5% Task value −6.7% Self-efficacy	x1.5 < Task value	−8.3% Task value −3.6 Self-efficacy 5.2 Test anxiety	x1.7 < Task value x1.6 < Test anxiety

Note. N/A = Not applicable.

**Table 5:** Recommendations for strengthening motivational beliefs about learning in victims and bullies of bullying and cyberbullying.

		Victims	Bullies
Students	Bullying	Enhance self-efficacy through strategies that build confidence in academic abilities. For girls, focus on reducing fatalism and strengthening their perception of competence. Implement stress management techniques to alleviate test anxiety, fostering a sense of security in learning. This is particularly important for girls, who exhibit higher levels of anxiety. Connect task value to meaningful learning experiences, emphasizing its relevance for future academic and professional success. For boys, highlight the relationship between effort and long-term achievement.	Implement programs that enhance task value and self-efficacy, fostering a positive outlook on learning. For boys, reinforce the role of effort in academic achievement. Develop emotional regulation strategies to prevent aggression and impulsivity from interfering with academic motivation. Promote perspective-taking and empathy, linking them to greater overall satisfaction.
	Cyberbullying	Promote digital resilience and the development of coping strategies to safeguard academic motivation against exposure to online harassment. For girls, strengthen social support networks to mitigate the internalization of negative experiences and their impact on self-efficacy. Train students to recognize and manage cyberbullying to minimize its effects on their perception of learning and motivation.	Promote digital literacy with a focus on the positive use of technology to sustain a constructive attitude toward learning. For girls, address the link between relational aggression and long-term academic demotivation. Implement strategies to help students recognize how their actions impact not only others but also their own perception of learning.
Educators	Bullying	Implement active learning methodologies that encourage participation and build confidence in the classroom, fostering self-efficacy among affected students. Develop strategies to reduce test anxiety, incorporating formative assessments and relaxation techniques. Design activities that reinforce task value, demonstrating its real-world applicability. For boys, emphasize the connection between effort and future success.	Integrate content on the impact of aggression on academic motivation, fostering self-control and self-regulation. Emphasize the development of long-term academic goals, strengthening students’ perception of control over their learning. Establish norms that reinforce mutual respect and positive coexistence, creating an environment that supports motivation.
	Cyberbullying	Develop classroom intervention strategies to ensure that the impact of cyberbullying does not undermine motivation and perception of learning. For girls, create spaces where they can express their concerns.	Incorporate emotional management and digital self-regulation tools into the curriculum, promoting responsible use of social media. For girls, address the connection between online aggression and declining interest in learning.
Family	Bullying	Foster a supportive home environment that strengthens self-efficacy and task value, ensuring that children develop confidence in their academic abilities. For girls, focus on building a positive academic identity. Implement communication strategies to help manage test anxiety and academic stress. For boys, emphasize the importance of verbalizing their emotions.	Reinforce the positive impact of learning at home, linking it to personal and academic goals. For boys, encourage greater engagement in their own academic development. Model emotional self-regulation and conflict resolution strategies to prevent disengagement from learning from becoming associated with patterns of aggression.
	Cyberbullying	Monitor and educate students on the impact of digital harassment on academic motivation, promoting healthy technology use. For both boys and girls, remain vigilant for signs of isolation and shifts in their perception of academic abilities.	Set clear boundaries for digital device use and promote a culture of respect in virtual environments. For both boys and girls, reinforce education on the consequences of cyberbullying and its impact on academic motivation.

## Data Availability

The data supporting the findings of this study are not publicly available due to privacy and ethical restrictions. This research is part of a larger investigation involving multiple researchers, and data confidentiality was a key requirement for participant involvement. To ensure compliance with ethical guidelines and to protect the privacy of all participants, the data cannot be shared.

## References

[B1-ejihpe-15-00093] Aparisi D., Delgado B., Bo R. M., Martínez-Monteagudo M. C. (2021). Relationship between cyberbullying, motivation and learning strategies, academic performance, and the ability to adapt to university. International Journal of Environmental Research and Public Health.

[B2-ejihpe-15-00093] Bacon P., Lord R. N. (2021). The impact of physically active learning during the school day on children’s physical activity levels, time on task and learning behaviours and academic outcomes. Health Education Research.

[B3-ejihpe-15-00093] Baharvand P., Nejad E. B., Karami K., Amraei M. (2021). A review study of the role of socioeconomic status and its components in children’s health. Global Journal of Medical, Pharmaceutical, and Biomedical Update.

[B4-ejihpe-15-00093] Beatson N. J., Berg D. A. G., Smith J. K. (2020). The influence of self-efficacy beliefs and prior learning on performance. Accounting and Finance.

[B5-ejihpe-15-00093] Bork-Hüffer T., Mahlknecht B., Kaufmann K. (2020). (Cyber)Bullying in schools–when bullying stretches across cON/FFlating spaces. Children’s Geographies.

[B6-ejihpe-15-00093] Burns E. C., Martin A. J., Collie R. J. (2018). Adaptability, personal best (PB) goals setting, and gains in students’ academic outcomes: A longitudinal examination from a social cognitive perspective. Contemporary Educational Psychology.

[B7-ejihpe-15-00093] Cañas E., Estévez E., Martínez-Monteagudo M. C., Delgado B. (2020). Emotional adjustment in victims and perpetrators of cyberbullying and traditional bullying. Social Psychology of Education.

[B8-ejihpe-15-00093] Compton L., Campbell M. A., Mergler A. (2014). Teacher, parent and student perceptions of the motives of cyberbullies. Social Psychology of Education.

[B9-ejihpe-15-00093] Cosma A., Bjereld Y., Elgar F. J., Richardson C., Bilz L., Craig W., Augustine L., Molcho M., Malinowska-Cieślik M., Walsh S. D. (2022). Gender differences in bullying reflect societal gender inequality: A multilevel study with adolescents in 46 countries. Journal of Adolescent Health.

[B10-ejihpe-15-00093] Delgado B., Escortell R., Martínez-Monteagudo M. C., Ferrández-Ferrer A., Sanmartín R. (2019). Cyberbullying, self-concept and academic goals in childhood. The Spanish Journal of Psychology.

[B11-ejihpe-15-00093] Del Rey R., Casas J. A., Ortega-Ruiz R., Schultze-Krumbholz A., Scheithauer H., Smith P., Thompson F., Barkoukis V., Tsorbatzoudis H., Brighi A., Guarini A., Pyzalski J., Plichta P. (2015). Structural validation and cross-cultural robustness of the European cyberbullying intervention project questionnaire. Computers in Human Behavior.

[B12-ejihpe-15-00093] Deol Y., Lashai M. (2022). Impact of cyberbullying on adolescent mental health in the midst of pandemic—Hidden crisis. European Psychiatry.

[B13-ejihpe-15-00093] El Zaatari W., Maalouf I. (2022). How the bronfenbrenner bio-ecological system theory explains the development of students’ sense of belonging to school?. SAGE Open.

[B14-ejihpe-15-00093] Eyuboglu M., Eyuboglu D., Pala S. C., Oktar D., Demirtas Z., Arslantas D., Unsal A. (2021). Traditional school bullying and cyberbullying: Prevalence, the effect on mental health problems and self-harm behavior. Psychiatry Research.

[B15-ejihpe-15-00093] Fateel M. J., Mukallid S., Arora B. (2021). The interaction between socioeconomic status and preschool education on academic achievement of elementary school students. International Education Studies.

[B16-ejihpe-15-00093] Galán-Arroyo C., Flores-Ferro E., Castillo-Retamal F., Rojo-Ramos J. (2024). Motor self-efficacy and physical education in school bullying. Frontiers in Psychology.

[B17-ejihpe-15-00093] Geng J., Wang Y., Wang P., Zeng P., Lei L. (2022). Gender differences between cyberbullying victimization and meaning in life: Roles of fatalism and self-concept clarity. Journal of Interpersonal Violence.

[B18-ejihpe-15-00093] Hinduja S., Patchin J. W. (2017). Cultivating youth resilience to prevent bullying and cyberbullying victimization. Child Abuse and Neglect.

[B19-ejihpe-15-00093] Hosozawa M., Bann D., Fink E., Elsden E., Baba S., Iso H., Patalay P. (2021). Bullying victimisation in adolescence: Prevalence and inequalities by gender, socioeconomic status and academic performance across 71 countries. EClinicalMedicine 41.

[B20-ejihpe-15-00093] Koenka A. C. (2020). Academic motivation theories revisited: An interactive dialog between motivation scholars on recent contributions, underexplored issues, and future directions. Contemporary Educational Psychology.

[B21-ejihpe-15-00093] Kryshko O., Fleischer J., Waldeyer J., Wirth J., Leutner D. (2020). Do motivational regulation strategies contribute to university students’ academic success?. Learning and Individual Differences.

[B22-ejihpe-15-00093] Kwon S., Welch S., Mason M. (2020). Physical education environment and student physical activity levels in low-income communities. BMC Public Health.

[B23-ejihpe-15-00093] Lepinet U., Tanniou J., Communier E. C., Créach V., Beaumont M. (2023). Impact of anthropometric data and physical activity level on the closed kinetic chain upper extremity stability test (CKCUEST) score: A cross-sectional study. Physical Therapy in Sport.

[B24-ejihpe-15-00093] Li D., Wang D., Zou J., Li C., Qian H., Yan J., He Y. (2023). Effect of physical activity interventions on children’s academic performance: A systematic review and meta-analysis. European Journal of Pediatrics.

[B25-ejihpe-15-00093] Li L., Chen X., Li H. (2020). Bullying victimization, school belonging, academic engagement and achievement in adolescents in rural China: A serial mediation model. Children and Youth Services Review.

[B26-ejihpe-15-00093] Liu Y., Hau K. T., Liu H., Wu J., Wang X., Zheng X. (2020). Multiplicative effect of intrinsic and extrinsic motivation on academic performance: A longitudinal study of Chinese students. Journal of Personality.

[B27-ejihpe-15-00093] Liu Y., Yu X., An F., Wang Y. (2023). School bullying and self-efficacy in adolescence: A meta-analysis. Journal of Adolescence.

[B28-ejihpe-15-00093] Martínez-López E. J., De La Torre-Cruz M., Suarez-Manzano S., Ruiz-Ariza A. (2018). Analysis of the effect size of overweight in muscular strength tests among adolescents: Reference values according to sex, age, and body mass index. Journal of Strength and Conditioning Research.

[B29-ejihpe-15-00093] Martínez-Monteagudo M. C., Delgado B., García-Fernández J. M., Ruíz-Esteban C. (2020). Cyberbullying in the university setting. relationship with emotional problems and adaptation to the university. Frontiers in Psychology.

[B30-ejihpe-15-00093] Montero-Carretero C., Barbado D., Cervelló E. (2020). Predicting bullying through motivation and teaching styles in physical education. International Journal of Environmental Research and Public Health.

[B31-ejihpe-15-00093] Montolio D., Taberner P. A. (2021). Gender differences under test pressure and their impact on academic performance: A quasi-experimental design. Journal of Economic Behavior and Organization.

[B32-ejihpe-15-00093] Obregón-Cuesta A. I., Mínguez-Mínguez L. A., León-del-Barco B., Mendo-Lázaro S., Fernández-Solana J., González-Bernal J. J., González-Santos J. (2022). Bullying in adolescents: Differences between gender and school year and relationship with academic performance. International Journal of Environmental Research and Public Health.

[B33-ejihpe-15-00093] O’Higgins Norman J. (2020). Tackling bullying from the inside out: Shifting paradigms in bullying research and interventions. International Journal of Bullying Prevention.

[B34-ejihpe-15-00093] Okumu M., Kim Y. K., Sanders J. E., Makubuya T., Small E., Hong J. S. (2020). Gender-specific pathways between face-to-face and cyber bullying victimization, depressive symptoms, and academic performance among U.S. adolescents. Child Indicators Research.

[B35-ejihpe-15-00093] Ortega-Ruiz R., Del Rey R., Casas J. A. (2016). Evaluar el bullying y el cyberbullying validación española del EBIP-Q y del ECIP-Q. Psicologia Educativa.

[B36-ejihpe-15-00093] Peled Y. (2019). Cyberbullying and its influence on academic, social, and emotional development of undergraduate students. Heliyon.

[B37-ejihpe-15-00093] Petrigna L., Thomas E., Brusa J., Rizzo F., Scardina A., Galassi C., Lo Verde D., Caramazza G., Bellafiore M. (2022). Does learning through movement improve academic performance in primary schoolchildren? A systematic review. Frontiers in Pediatrics.

[B38-ejihpe-15-00093] Pintrich P. R. R., Smith D., Garcia T., McKeachie W. (1991). A manual for the use of the motivated strategies for learning questionnaire (MSLQ). Ann Arbor. Michigan.

[B39-ejihpe-15-00093] Poh B. K., Lee S. T., Yeo G. S., Tang K. C., Noor Afifah A. R., Siti Hanisa A., Parikh P., Wong J. E., Ng A. L. O., Norimah A. K., Ruzita A. T., Budin S. B., Siti Haslinda M. D., Ismail M. N., Rahman J., Kamaruddin N. A., Nik Shanita S., Chin Y. S., Wee B. S., Jamil N. A. (2019). Low socioeconomic status and severe obesity are linked to poor cognitive performance in Malaysian children. BMC Public Health.

[B40-ejihpe-15-00093] Potard C., Kubiszewski V., Combes C., Henry A., Pochon R., Roy A. (2022). How adolescents cope with bullying at school: Exploring differences between pure victim and bully-victim roles. International Journal of Bullying Prevention.

[B41-ejihpe-15-00093] Prochaska J. J., Sallis J. F., Long B. (2001). A physical activity screening measure for use with adolescents in primary care. Archives of Pediatrics & Adolescent Medicine.

[B42-ejihpe-15-00093] Przybylski A. K., Bowes L. (2017). Cyberbullying and adolescent well-being in England: A population-based cross-sectional study. The Lancet Child and Adolescent Health.

[B43-ejihpe-15-00093] Raine L. B., Kao S. C., Drollette E. S., Pontifex M. B., Pindus D., Hunt J., Kramer A. F., Hillman C. H. (2020). The role of BMI on cognition following acute physical activity in preadolescent children. Trends in Neuroscience and Education.

[B44-ejihpe-15-00093] Rejeb A., Rejeb K., Zrelli I., Süle E. (2024). Tracing knowledge diffusion trajectories in the research field of cyberbullying. Heliyon.

[B45-ejihpe-15-00093] Roos A. L., Goetz T., Krannich M., Donker M., Bieleke M., Caltabiano A., Mainhard T. (2023). Control, anxiety and test performance: Self-reported and physiological indicators of anxiety as mediators. British Journal of Educational Psychology.

[B46-ejihpe-15-00093] Rusillo-Magdaleno A., De la Torre-Cruz M. J., Ruiz-Ariza A., Suárez-Manzano S. (2024). Association of high levels of bullying and cyberbullying with test anxiety in boys and girls aged 10 to 16 years. Education Sciences.

[B47-ejihpe-15-00093] Samara M., Nascimento B. D. S., El-Asam A., Hammuda S., Khattab N. (2021). How can bullying victimisation lead to lower academic achievement? A systematic review and meta-analysis of the mediating role of cognitive-motivational factors. International Journal of Environmental Research and Public Health.

[B48-ejihpe-15-00093] Seum T., Meyrose A. K., Rabel M., Schienkiewitz A., Ravens-Sieberer U. (2022). Pathways of parental education on children’s and adolescent’s body mass index: The mediating roles of behavioral and psychological factors. Frontiers in Public Health.

[B49-ejihpe-15-00093] Song J., Chung Y. (2020). Reexamining the interaction between expectancy and task value in academic settings. Learning and Individual Differences.

[B50-ejihpe-15-00093] Steinmayr R., Weidinger A. F., Schwinger M., Spinath B. (2019). The importance of students’ motivation for their academic achievement-replicating and extending previous findings. Frontiers in Psychology.

[B51-ejihpe-15-00093] Tantoh M. C. (2023). Parental Level of Education and its Implications of their Expectations towards their Children Academic Performance. International Journal of Psychology and Cognitive Education.

[B52-ejihpe-15-00093] Thorsen C., Yang Hansen K., Johansson S. (2021). The mechanisms of interest and perseverance in predicting achievement among academically resilient and non-resilient students: Evidence from Swedish longitudinal data. British Journal of Educational Psychology.

[B53-ejihpe-15-00093] Urhahne D., Wijnia L. (2023). Theories of motivation in education: An integrative framework. Educational Psychology Review.

[B54-ejihpe-15-00093] Urruticoechea A., Oliveri A., Vernazza E., Giménez-Dasí M., Martínez-Arias R., Martín-Babarro J. (2021). The relative age effects in educational development: A systematic review. International Journal of Environmental Research and Public Health.

[B55-ejihpe-15-00093] Valera-Pozo M., Flexas A., Servera M., Aguilar-Mediavilla E., Adrover-Roig D. (2021). Long-term profiles of bullying victims and aggressors: A retrospective study. Frontiers in Psychology.

[B56-ejihpe-15-00093] Van Vu T., Scharmer A. L., van Triest E., van Atteveldt N., Meeter M. (2024). The reciprocity between various motivation constructs and academic achievement: A systematic review and multilevel meta-analysis of longitudinal studies. Educational Psychology.

[B57-ejihpe-15-00093] Vismara M., Girone N., Conti D., Nicolini G., Dell’Osso B. (2022). The current status of cyberbullying research: A short review of the literature. Current Opinion in Behavioral Sciences.

[B58-ejihpe-15-00093] Wassenaar T. M., Wheatley C. M., Beale N., Nichols T., Salvan P., Meaney A., Atherton K., Diaz-Ordaz K., Dawes H., Johansen-Berg H. (2021). The effect of a one-year vigorous physical activity intervention on fitness, cognitive performance and mental health in young adolescents: The Fit to Study cluster randomised controlled trial. International Journal of Behavioral Nutrition and Physical Activity.

[B59-ejihpe-15-00093] Wirthwein L., Sparfeldt J. R., Heyder A., Buch S. R., Rost D. H., Steinmayr R. (2020). Sex differences in achievement goals: Do school subjects matter?. European Journal of Psychology of Education.

[B60-ejihpe-15-00093] Worick C. E., Usher E. L., Osterhage J., Love A. M. A., Keller P. S. (2023). Self-efficacy for self-regulated learning mediates association between implicit theories of willpower and learning strategies. The Journal of Experimental Education.

[B61-ejihpe-15-00093] Yang S., Wang W. (2022). The role of academic resilience, motivational intensity and their relationship in EFL learners’ academic achievement. Frontiers in Psychology.

[B62-ejihpe-15-00093] Zhu C., Huang S., Evans R., Zhang W. (2021). Cyberbullying among adolescents and children: A comprehensive review of the global situation, risk factors, and preventive measures. Frontiers in Public Health.

[B63-ejihpe-15-00093] Zsila Á., Urbán R., Griffiths M. D., Demetrovics Z. (2019). Gender differences in the association between cyberbullying victimization and perpetration: The role of anger rumination and traditional bullying experiences. International Journal of Mental Health and Addiction.

